# Response to Martin and colleagues: mitochondria do not boost the bioenergetic capacity of eukaryotic cells

**DOI:** 10.1186/s13062-018-0228-3

**Published:** 2018-11-29

**Authors:** Michael Lynch, Georgi K. Marinov

**Affiliations:** 1 Biodesign Center for Mechanisms of Evolution, Arizona State University, Tempe, AZ 85287 USA; 2 Department of Genetics, Stanford University School of Medicine, Stanford, CA 94305 USA

**Keywords:** Bioenergetics, Cell size, Eukaryogenesis, Mitochondrion, Ribosomes

## Abstract

A recent paper by (Gerlitz et al., Biol Direct 13:21, 2018) questions the validity of the data underlying prior analyses on the bioenergetics capacities of cells, and continues to promote the idea that the mitochondrion endowed eukaryotic cells with energetic superiority over prokaryotes. The former point has been addressed previously, with no resultant changes in the conclusions, and the latter point remains inconsistent with multiple lines of empirical data.

## Text

Three months prior to Gerlitz et al. [1], we published a reevaluation and more thorough annotation of the data sources to which they refer [9]. Prior to embarking on their mission, these authors could have sought clarification by communicating directly with us, but did not do so. Our reanalysis lead to a slightly improved statistical fit of the ribosome number – cell volume scaling relationship, although not significantly so. Contrary to the claims of Gerlitz et al. [1], there were no systematic errors. Contrary to the claims of Gerlitz et al. [1] the original version of our paper is available at eLife, at https://elifesciences.org/articles/20437v1.

The senior author of the comment to which we are responding (WFM) has argued that the establishment of the mitochondrion endowed eukaryotes with extraordinary energetic capacity, without which the complexity of eukaryotic cells would be impossible [3–5, 10]. Our work suggests otherwise [6, 8]. This is not to say that mitochondrial endosymbiosis was not a watershed event in eukaryotic evolution, but a diversity of analyses consistently indicate that there is no quantum leap in the energetic capacity of eukaryotes relative to prokaryotes, but instead continuity of allometric scaling within and between phylogenetic groups.

To put things into context, our initial interest in cellular bioenergetics was motivated by the unsolved problem of the amount of energy required to build a cell and to run its various genes [6]. While performing our analyses, we realized that the data failed to support the hypothesis of Lane and Martin [3] that the mitochondrion precipitated a bioenergetic revolution said to enable a 200,000-fold increase in gene number (the source of this factor remains unclear). Lane and Martin [5] objected, raising the point that we had neglected ribosomes in ways that would somehow affect the legitimacy of our conclusions. We addressed the ribosome issue in Lynch and Marinov [8, 9], showing that it in no way alters our prior conclusions. Motivated by ideas generated in our previous response to Lane and Martin [7], we also considered the substantial investment that eukaryotes make in lipid bilayers, as well as the abundance of ATP synthase per cell. These analyses also support the contention that the mitochondrion has not endowed eukaryotes with extraordinary energetic capacity, contrary to the conclusions in Lane and Martin [3].

Rather than providing scientific arguments relevant to the debate about mitochondria and the limitations of eukaryotic bioenergetics, Gerlitz et al. [1] question the existence of the data upon which our analyses were based. Their contention is that many of the cell-volume data in Lynch and Marinov [8] do not exist. Confusion was caused by our failure to note that the citations for these data were contained in tables other than the specific one that Gerlitz et al. [1] focused on, which pertained to ribosome content. These issues are clarified in Lynch and Marinov [9].

Two additional points of concern in Gerlitz et al. [1] merit comment. First, they note that the cellular/physiological data from bacteria and eukaryotes are not drawn from the same distribution. No one has argued otherwise. What we have emphasized is the continuity in the scaling relationships of such data with cell size (Figures 1a, 2, and 4 in Lynch and Marinov [6]; Figures 1 and 2 in Lynch and Marinov [8]). Continuity in scaling refers to the absence of a discontinuous shift in an allometric relationship between two measures, which does not depend on complete continuity in distribution.

Second, Gerlitz et al. [1] repeat the assertion that we have misinterpreted the bioenergetics claims in Lane and Martin [3]. However, direct quotes from the latter show that this is not the case ([7]; see also the quotes in the Response to Reviewers in Lynch and Marinov [8]). Lane and Martin’s definition of energetic capacity, the ratio of the total energy assimilated per number of genes, has no meaningful connection with energetics or evolution. Their metric is analogous to rating the fuel economy of car by dividing the volume of the gas tank by the number of parts. Under this view, an embellished vehicle with so many power-demanding bells and whistles that no fuel remains for generating mileage would be regarded as superior.

Martin’s current attention to detail is welcome. Graur and Martin [2] provide a commentary on how chains of uncertainties sometimes morph into facts. Yet, in the primary paper of contention [3], which Martin views as established fact, it is stated that “There is an appreciable range of uncertainty in measurement for both cell mass and metabolic rates for microbes: values differing by one or two orders of magnitude might not be meaningfully different.” In a recent paper attempting to bolster the original claims of Lane and Martin [3], Martin [10] states that “In evolution there are no facts, there are only observations and their interpretation.”

As in all sciences, the goal of evolutionary biology is to establish the material basis of facts and to settle disagreements via rational debate. In performing our analyses, it has been essential to rely on data from various sources, presumably with various levels of accuracy, and wherever possible we have attempted to use average observations over independent studies. The strong scaling relationships presented in Lynch and Marinov [6, 8] are inconsistent with a substantial error component in the data analyses, as this would greatly reduce the significance of the regressions and diminish the allometric coefficients relative to expectations under isometry. Indeed, the analysis of Gerlitz et al. [1], based on a highly reduced data set strongly supports our contention that there is no quantum leap in translational capacity in the bridge between prokaryotes and eukaryotes. We thank the authors for their supportive analyses.

## References

[1] Gerlitz M, Knopp M, Kapust N, Xavier JC, Martin WF (2018). Elusive data underlying debate at the prokaryote-eukaryote divide. Biol Direct.

[2] Graur D, Martin W (2004). Reading the entrails of chickens: molecular timescales of evolution and the illusion of precision. Trends Genet.

[3] Lane N, Martin W (2010). The energetics of genome complexity. Nature.

[4] Lane N, Martin WF (2015). Eukaryotes really are special, and mitochondria are why. Proc Natl Acad Sci U S A.

[5] Lane N, Martin WF (2016). Mitochondria, complexity, and evolutionary deficit spending. Proc Natl Acad Sci U S A.

[6] Lynch M, Marinov GK (2015). The bioenergetic costs of a gene. Proc Natl Acad Sci U S A.

[7] Lynch M, Marinov GK (2016). Reply to Lane and Martin: mitochondria do not boost the bioenergetic capacity of eukaryotic cells. Proc Natl Acad Sci U S A.

[8] Lynch M, Marinov GK (2017). Membranes, energetics, and evolution across the prokaryote-eukaryote divide. ELife.

[9] Lynch M, Marinov GK (2018). Correction: membranes, energetics, and evolution across the prokaryote-eukaryote divide. Elife.

[10] Martin WF (2017). Symbiogenesis, gradualism, and mitochondrial energy in eukaryote evolution. Period Biol.

